# Dynamic Threshold Cable-Stayed Bridge Health Monitoring System Based on Temperature Effect Correction

**DOI:** 10.3390/s23218826

**Published:** 2023-10-30

**Authors:** Dongmei Tan, Tai Guo, Hao Luo, Baifeng Ji, Yu Tao, An Li

**Affiliations:** 1 School of Civil Engineering and Architecture, Wuhan University of Technology, Wuhan 430070, China; smiledongmei@whut.edu.cn (D.T.); gt32882323@163.com (T.G.); lh3390864306@whut.edu.cn (H.L.); taoyu123456@whut.edu.cn (Y.T.); lian320327@whut.edu.cn (A.L.); 2 Hainan Institute, Wuhan University of Technology, Sanya 572000, China

**Keywords:** health monitoring, early warning, cable-stayed bridge, dynamic threshold

## Abstract

The early health warning of a cable-stayed bridge is of great significance for discovering the abnormal condition of the structure, eliminating the risk factors, and ensuring the normal operation of the bridge in order to set a reasonable safety monitoring threshold to ensure the safety warning and condition assessment of the bridge structure. A method of dynamic early warning by considering the temperature effect is adopted in this paper on the basis of the benchmark threshold. Based on the long-term deflection monitoring data of a bridge in Wuhan, the generalized Pareto distribution (GPD) extreme value analysis theory is used to set the benchmark threshold. Then, by constructing the seasonal autoregressive integrated moving average (SARIMA) long-span bridge temperature effect prediction model, the reference threshold is dynamically adjusted. Finally, it is compared with the traditional fixed threshold monitoring system. The results show that the dynamic threshold has stronger adaptability to the monitoring of cable-stayed bridges and can also achieve effective monitoring of local mutations in other periods. Dynamic threshold early warning can reduce the shortcomings of traditional early warning methods such as underreporting and misreporting. At the same time, the GPD extreme value analysis theory overcomes the disadvantage that the extreme value information is not fully utilized. It has an important application value for bridge health monitoring.

## 1. Introduction

For decades, structural health monitoring (SHM) has become an important hotspot in civil engineering, machinery, automotive, and aerospace engineering [1,2,3]. With the development of bridge engineering, bridge construction technology is also constantly upgrading, and a variety of structural forms have appeared one after another. Long-span bridges such as the Tsing Ma Bridge in Hong Kong [4], the Great Belt Bridge in Denmark, the Messina Strait Bridge in Italy, and the Golden Ear Bridge in Canada have been put into use one after another. However, with the continuous extension of the service life of bridges, the external environmental conditions of bridges are also changing, such as wind load [5,6,7], ground temperature [8,9], temperature [10,11,12], humidity, and so on. Changes in these external environmental conditions will gradually reduce the durability and safety of bridge structures. Therefore, in order to keep abreast of the health status of bridges, many long-span bridges are equipped with health monitoring systems. By setting a reasonable early warning system, the potential bridge health risks can be effectively identified and reduced, thereby prolonging the service life of bridges.

The research of early warning systems has always been an important topic in the research of structural health monitoring (SHM) [13,14], which provides timely and reliable guidance for the management and maintenance of bridges. Chen et al. [15] carried out numerical and case studies on the early warning method of the immersed tunnel of the Hong Kong-Zhuhai-Macao Bridge. Based on the monitoring data of wavelet threshold denoising, the autoregressive integrated moving average (ARIMA) is used to predict future data. They developed a hierarchical early warning system to prove that the system can effectively classify detected anomalies. Zhao et al. [16,17] used the long short-term memory network to classify the vehicle-induced strain and used the reliability theory to determine the warning threshold with a guarantee rate of 95%. They also used the t-position scale distribution to determine the warning threshold for beam deflection caused by trains on steel truss arch railway bridges. Xin et al. [18] proposed a data-driven innovation method based on improved variational mode decomposition (IVMD) and conditional kernel density estimation (CKDE), which can accurately predict bridge deformation and is of great significance for bridge deformation warning. Selvakumaran et al. [19] used the improved Interferometric Synthetic Aperture Radar (InSAR) method to analyze the satellite observation scene before the collapse of the Tadcaster Bridge in the United Kingdom and proved that the method can be used as an effective early warning system for monitoring bridges at risk of erosion. Ding et al. [20] studied the correlation between train speed and bridge acceleration, and on this basis, an early warning method for lateral vibration anomaly identification was proposed by using wavelet decomposition and interval estimation theory. Kareem A. Eltouny et al. [21] proposed an uncertainty-aware early warning system that can provide near-real-time SHM. The system uses a deep composite encoder-decoder network that combines elements of convolutional neural networks, recurrent neural networks, and variational inference (VI) to provide an exponential distribution of damage. Biondi, F et al. [22] used a spaceborne synthetic aperture radar to monitor critical infrastructures, monitoring them effectively even in the case of clouds and very bad weather. Based on the modal attribute analysis, a complete damage early warning detection program is designed by using the micro-motion (m-m) estimation of key sites.

The temperature effect will lead to the phenomenon of underreporting and misreporting in the early warning system, and many scholars also consider this problem. For the early warning system, the influence of temperature effect needs to be considered. Xia et al. [12] pointed out that temperature response may cause missing and false alarms in structural damage warnings. Xu et al. [23] found that for the dynamic indicators in damage early warning, the fluctuation of indicators caused by temperature is more severe than that caused by damage.

Kromanis et al. [24] proposed a comprehensive evaluation of structural performance based on quasi-static measurement, which requires removing the temperature and vehicle-induced trends from the measurement time history of structural response and relying on the knowledge of vehicle load and its position on the bridge to predict vehicle response. Kullaa, J et al. [25] studied the difference between three different sources of variation in vibration-based structural health monitoring systems: environmental or operational effects, sensor failure, and structural damage. The separation of environmental or operational effects from the other two is based on the following assumption: the training data contain measurements under different environmental or operational conditions. The distinction between sensor failure and structural damage takes advantage of the fact that sensor failure is a local fault and structural damage is a global one. Xin et al. [26] proposed a new method for bridge structure deformation prediction by combining the Kalman filter, autoregressive integrated moving average model (ARIMA), and generalized autoregressive conditional heteroscedasticity (GARCH). Fan et al. [27] proposed an abnormal real-time dynamic early warning method for cable-stayed bridges based on deflection measurement considering the operating environment of bridges. The generalized Pareto distribution (GPD) model and finite element (FE) calculation are used to determine the basic warning threshold. 

Structural health monitoring is not limited to bridges. In the field of optimizing building thermal performance, Behnam Mobaraki et al. [28,29] proposed a Hyper Efficient Arduino Transmittance-meter (HEAT) based on low-cost technology to monitor U values. At the same time, through the results of an experimental activity, the influence of incorrectly positioned external sensors on the measurement accuracy of U values was evaluated. Min et al. [30] summarized optical fiber sensors for marine environmental and structural health monitoring to understand their basic sensing principles and various sensing applications, such as physical parameters, chemical parameters, and structural health monitoring. Tan et al. [31] studied the structural health monitoring (SHM) data collected based on underwater shield tunnels, coupled the spatial-temporal correlation with external loads through the autoencoder network (ATENet), and developed a real-time prediction model to predict the structural performance in the next 12 h.

By analyzing the current status of early warning methods of bridge health monitoring systems at home and abroad, it is found that the fluctuation of indicators caused by the environmental temperature will result in the phenomenon of omission and misreporting of the structural monitoring system. The influence of temperature effect on the early warning system should not be ignored, and the influence of temperature should be considered in the design of the early warning system. In previous studies, the adjustment of the warning threshold line is only updated regularly, in which the previous temperature effect data are used to adjust the current warning threshold line after a certain period. In order to solve this problem, a method is proposed in this paper to realize dynamic early warning by considering the temperature effect on the basis of the benchmark threshold by constructing the seasonal autoregressive integrated moving average (SARIMA) bridge temperature effect prediction model and combining it with the benchmark threshold determined by the theory of analyzing the extreme value of the generalized Pareto distribution.

In this paper, a dynamic threshold warning method based on temperature effect prediction is proposed. The method is verified by taking a long-span in-service cable-stayed bridge as an example. The rest of this article is organized as follows. Section 2 introduces the workflow of the Beidou navigation system, generalized Pareto extreme value analysis theory, and three fitting test methods, and then introduces the principle of the SARIMA model. In Section 3, the proposed method is introduced. Based on the health monitoring data of a bridge in Wuhan, the generalized Pareto extreme value analysis theory is used to predict the benchmark threshold. The SARIMA bridge temperature effect prediction model is constructed by separating the deflection data, and the temperature effect of the benchmark threshold is corrected to realize dynamic monitoring. Section 4 discusses the important findings, significance, and limitations of this study. Finally, concluding remarks are given in Section 5.

## 2. Methodology

### 2.1. Beidou Positioning Receiver

In terms of terminal equipment, single-frequency receivers can currently only receive B1 carrier signals which cannot effectively eliminate the impact of ionospheric delay, resulting in relatively low positioning accuracy. Dual-frequency receivers can receive both B1 and B2 carrier signals simultaneously and cancel out the delay error caused by the ionospheric electromagnetic wave signals based on observations of the two frequencies. Therefore, dual-frequency receivers can achieve higher positioning accuracy.

The basic principle of the Beidou satellite positioning system is to measure the distance between the known positions of the satellites and the user’s receiver and then determine the specific position of the receiver by combining data from multiple satellites.

(1) Theoretical Model

The Beidou navigation satellites continuously transmit navigation messages. The receiver extracts satellite ephemeris data and compares it with its own clock to obtain the time difference between the satellite and the user. The three-dimensional coordinates of the satellite at the time of the message transmission can be calculated using the satellite ephemeris data in the navigation message. The user’s three-dimensional coordinates can be calculated by solving a system of three equations based on the distance formula between two points in space, $d=\sqrt{a^{2}+b^{2}+c^{2}}$. Given the satellites’ coordinates $\left(x_{1},y_{1},z_{1}\right)$, $\left(x_{2},y_{2},z_{2}\right)$, and $\left(x_{3},y_{3},z_{3}\right)$, and the time differences between the satellites and receiver, t_1_, t_2_, and t_3_, the receiver’s three-dimensional coordinates $\left(x,y,z\right)$ can be determined from

(1) $$\left\{\begin{array}{c} {\left(x_{1}-x\right)}^{2}+{\left(y_{1}-y\right)}^{2}+{\left(z_{1}-z\right)}^{2}=c^{2}\cdot t_{1}^{2}\\ {\left(x_{2}-x\right)}^{2}+{\left(y_{2}-y\right)}^{2}+{\left(z_{2}-z\right)}^{2}=c^{2}\cdot t_{2}^{2}\\ {\left(x_{3}-x\right)}^{2}+{\left(y_{3}-y\right)}^{2}+{\left(z_{3}-z\right)}^{2}=c^{2}\cdot t_{3}^{2} \end{array}\right.$$

(2) Practical Model

In practical use, the Beidou navigation satellites use atomic clocks, which have an accuracy of picoseconds. However, receivers generally use quartz clocks, which have errors of positive and negative one-hundred-thousandths to ten-thousandths of a second per second. The satellite and receiver clocks cannot be completely synchronized when measuring the time difference between the satellite and the user. A fourth unknown variable, Δ*t*, the error between the receiver and satellite clocks, is introduced into the above equations to ensure positioning accuracy. By solving a system of four equations, the three-dimensional coordinates of the user and the accurate timing error value Δ*t* can be calculated, with Δ*t* used for precise time synchronization. Given the satellite coordinates $\left(x_{1},y_{1},z_{1}\right),\;\left(x_{2},y_{2},z_{2}\right),\;\left(x_{3},y_{3},z_{3}\right),\;and\;\left(x_{4},y_{4},z_{4}\right)$, and the time differences between the satellite and receiver, *t*_1_, *t*_2_, *t*_3_, and *t*_4_, the system of Equation (2) can be solved for Δ*t* and the receiver’s three-dimensional coordinates $\left(x,y,z\right)$.

(2) $$\left\{\begin{array}{c} \sqrt{{\left(x_{1}-x\right)}^{2}+{\left(y_{1}-y\right)}^{2}+{\left(z_{1}-z\right)}^{2}}=c\cdot \left(t_{1}-\Delta t\right)\\ \sqrt{{\left(x_{2}-x\right)}^{2}+{\left(y_{2}-y\right)}^{2}+{\left(z_{2}-z\right)}^{2}}=c\cdot \left(t_{2}-\Delta t\right)\\ \sqrt{{\left(x_{3}-x\right)}^{2}+{\left(y_{3}-y\right)}^{2}+{\left(z_{3}-z\right)}^{2}}=c\cdot \left(t_{3}-\Delta t\right)\\ \sqrt{{\left(x_{4}-x\right)}^{2}+{\left(y_{4}-y\right)}^{2}+{\left(z_{4}-z\right)}^{2}}=c\cdot \left(t_{4}-\Delta t\right) \end{array}\right.$$

The advantages of the Beidou navigation system in practical applications include high positioning accuracy, good anti-jamming capabilities, and wide coverage. However, factors such as ionospheric delay, multipath effects, and satellite clock errors can affect the system’s accuracy, and various error correction techniques and auxiliary positioning methods are often used to improve the system’s reliability and robustness.

### 2.2. Bridge Health Monitoring System

According to the system’s overall goal, the bridge safety monitoring system relies on various technologies, and its design ideas are as follows.

(1) Beidou high-precision positioning acquisition sensors and supporting equipment are installed in key parts of the bridge, and the surrounding Beidou reference station is built to establish a stable monitoring system to continuously collect and analyze the operation status and deformation information of the bridge.
(2) Based on the optical fiber communication mode, transmit the collected bridge geometric spatial data and the stable reference coordinates of the Beidou reference station to the bridge cloud computer room for data processing and storage backup.
(3) Implement perfect software functions, intuitive data display and publishing functions, convenient query functions, and statistical and automatic report functions to ensure the scalability and stability of the hardware system.
(4) The data input interface of the bridge Internet of Things safety monitoring system combines various types of sensor monitoring data with Beidou safety monitoring data to control the operational safety of the bridge by combining macroscopic deformation and microscopic structural data.
(5) Provide exclusive cloud services for the bridge to assure the operating environment and security of data processing and analysis software, realize the storage and backup of bridge data, form a bridge operation big data resource pool, and ensure the effective operation of the entire safety monitoring system.

**Figure 1:**
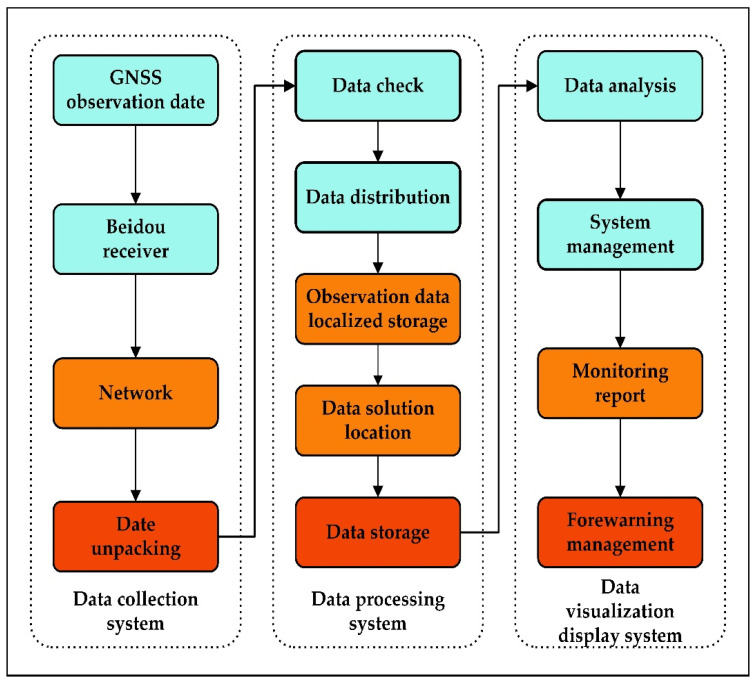
Bridge Health Monitoring System-specific diagram.

A diagram illustrating these specific processes is provided in Figure 1.

### 2.3. Base Threshold

Extreme value statistics methods mainly consist of interval extreme value and threshold-exceeding methods. The interval extreme value method divides the monitoring data $\left\{X_{1},X_{2},\;X_{3}\cdots \cdots X_{n}\right\}$ into N intervals and takes each interval’s maximum value as the sample for extreme value statistics. The second-largest value in a particular interval may be larger than the maximum value in other intervals, leading to insufficient utilization of the extreme value information in the data. The threshold-exceeding method selects all data higher than a specified limit (threshold) as the sample for extreme value statistics, overcoming the disadvantages of the interval extreme value method. However, threshold selection plays a decisive role in extreme value estimation.

#### 2.3.1. Generalized Extreme Value Distribution 

Assuming that the random variables $X_{1},X_{2},\;X_{3}\cdots \cdots ,X_{n}$ are independent and follow the same distribution F(x) and taking the maximum value $M_{n}=max\{X_{1},X_{2},X_{3}\cdots \cdots {,X}_{n}\}$, then

(3) $$P(M_{n}\leq x)=P(X_{1}\leq x,X_{2}\leq x,\cdots ,X_{n}\leq x)={\;[F\left(x\right)]}^{n}$$

If F(x) is known, the distribution function of the maximum value can be calculated based on the above equation, but F(x) is generally unknown in practical engineering applications.

According to the extreme value theorem, if there exists $a_{n}>0,b_{n}\in R$, a non-degenerate distribution function H(x), and

(4) Pn→∞lim(Mn−bnan≤x)=H(x)

when Equation (4) is satisfied, H(x) is referred to as the extreme value distribution, and H(x) must belong to one of the following three types [32,33]:

(1) Gumbel Distribution Type

$$H_{1}(x)=\exp\{{-e}^{-x}\},-\infty <x<+\infty$$

(2) Frechet Distribution Type

$$H_{2}(x;\alpha )=\left\{\begin{array}{c} \;\;\;\;\;\;\;0\;\;\;\;\;\;,\;x\leq 0\\ \exp\{{-(-x)}^{\alpha }\},x>0 \end{array}\right.\;\alpha >0$$

(3) Weibull Distribution Type

$$H_{3}(x;\alpha )=\left\{\begin{array}{c} \exp\{-{\left(-x\right)}^{\alpha }\},x\leq 0\\ \;\;\;\;\;\;\;\;1\;\;\;\;\;\;,\;x\leq 0\; \end{array}\right.\alpha >0$$

The three extreme value distributions can be unified as follows:

(5) $$H\left(x;\mu ,\sigma ,\xi \right)=\exp\left\{{-\left(1+\xi \frac{x-\mu }{\sigma }\right)}^{-\frac{1}{\xi }}\right\},1+\xi \frac{x-\mu }{\sigma }>0;\sigma >0;\mu ,\xi \in R$$

The above equation is called the generalized extreme value (GEV) distribution, where μ is the location parameter, σ is the scale parameter, and ξ is the shape parameter.

#### 2.3.2. Generalized Pareto Distribution(GPD)

(1) Mean Excess Function

Assume that $X_{1},\;X_{2},\;X_{3}\cdots \cdots X_{n}$ are independent random variables with the same distribution F(x), and a fixed large value u is used as a threshold. If $X_{i}>u$, it is called an exceedance, and $y=X_{i}-u$ is the corresponding excess. Therefore, the distribution function of excess is [34]:

(6) $$F_{u}(y)=P(\left(X-u\leq y|X>u\right)=\frac{P(u\leq X\leq y+u)}{P(X>u)}=\frac{F(y+u)-F(u)}{1-F(u)}$$

The probability density function of an excess amount is:

(7) $$f_{u}(y)=\frac{f(y+u)}{1F(u)},y\geq 0$$

The cumulative distribution function of an excess threshold is:

(8) $$F_{\left[u\right]}\left(x\right)=P\left(X\leq x|X>u\right)=\frac{F\left(x\right)-F\left(u\right)}{1-F\left(u\right)},\;x\geq u$$

The probability density function of an excess threshold is:

(9) $$f_{\;[u]}(x)=\frac{f(x)}{1-F(u)},\;x\geq u$$

The definition of *e(u)* is the average excess function of *X*:

(10) $$e(u)=E(X-u\left|X>u)\right.$$

(2) Return level

One of the main purposes of extreme value analysis is to estimate the quantile $x_{p}$ at a certain guarantee level and to use $x_{p}$ as the reference threshold u(T). Suppose $X_{1},\;X_{2},\;X_{3}\cdots \cdots X_{n}$ are independent random variables with the same distribution F(x), and for a certain threshold u, the excess event $\left\{X_{i}>u\right\}$ is considered. The so-called *T*-year return level u(T) requires that the average number of times exceeding the reference threshold u(T) in *T* years of observation is 1, where $X_{i}$ is the observed value in the *i*th year. The equation is:

(11) $$u(T)=F^{-1}(1-1/T)$$

The threshold $u\left(T\right)$ is the (1−1/T) quantile of F(x). Because $P(X_{i}>u(T))=1-F(u(T))=1/T$, the *T*-year return level u(T) represents the probability that a maximum observed value exceeds u(T) is 1/T. 

Let $\tau_{1}=min\left\{m:X_{m}>u\left(T\right)\right\}$ be the first time that the threshold u(T) is exceeded. Then, the *r*^th^ time that the threshold is exceeded is given by $\tau_{r}=min\{m>\tau_{r-1}:X_{m}>u(T)\}$, for r>1:

(12) $$P(\tau_{1}=k)=P(X_{1}\leq u(T),\cdots ,X_{k-1}\leq u(T),X_{k}>u(T))=q{\left(1-q\right)}^{k-1},k=1,2,3,\cdots$$

where q=1−F(u(T)).

The reference threshold estimated in this paper corresponds to a quantile with a 95% probability of occurrence within 100 years and can be expressed as:

(13) $$P(\tau_{1}\leq 100)\leq 0.05$$

From Equations (6)–(10), It is evident that:

(14) $$P(\tau_{1}\leq k)=q\sum_{i=1}^{k}{\left(1-q\right)}^{i-1}=1-{\left(1-q\right)}^{k},k=1,2,3\cdots$$

By substituting Equation (14) into Equation (13), *q* can be obtained. The reference threshold $u\left(T\right)$ can be obtained from q=1/T and Equation (11).

(3) Generalized Pareto Distribution

In practical engineering applications, the distribution function F(x) of random variables $X_{1},\;X_{2},\;X_{3}\cdots \cdots X_{n}$ is generally unknown, so the distribution function $F_{\;[u]}(x)$ of their excesses above a threshold *u* is also unknown. When the threshold is sufficiently large, Pickands gives the asymptotic distribution of the excess distribution function under the condition of unknown F(x), which is called the generalized Pareto distribution (GPD) [35]:

(15) $$(x;\mu ,\sigma ,\xi )=1-{\left(1+\xi \frac{x-\mu }{\sigma }\right)}^{1/\xi },x\geq \mu ,\sigma >0,1+\xi \frac{x-\mu }{\sigma }>0$$

where μ is the location parameter, σ is the scale parameter, and ξ is the shape parameter. The GPD is used to model the distribution of exceedances or exceedance probabilities by fitting the tail data of the random variable.

(4) Parameter estimation of generalized Pareto distribution

The key to GPD parameter estimation is the location parameter, i.e., the threshold. When the chosen threshold is too large, relatively few data points exceed it, leading to a large sample variance. By contrast, if the threshold is too small, data distribution exceeding the threshold may differ significantly from the GPD. The mean excess plot is a commonly used method for threshold determination.

The mean excess plot method involves establishing the relationship between the average excess function e(u) and the threshold *u* to select the optimal threshold. For data that follow the GPD, the average excess function can be represented as:

(16) $$(u)=E(X-u\left|X>u\right.)=\frac{\sigma_{u}+\xi u}{1-\xi },\xi <1$$

In Equation (16), *u* is the threshold, ξ is the shape parameter, and $\sigma_{u}$ is the scale parameter corresponding to the threshold *u*. From Equation (16), e(u) has a linear relationship with *u*. For the dataset $X_{1},\;X_{2},\;X_{3}\cdots \cdots X_{n}$, the empirical estimate of e(u) is:

(17) $$e(u)=\frac{1}{N_{u}}\sum_{i=1}^{N_{u}}\left(X_{i}-u\right),X_{i}>u$$

where $N_{u}$ is the number of values in the dataset exceeding the threshold u.

For a threshold $u_{0}$, the excesses over $u_{0}$ are approximately distributed as the generalized Pareto distribution with shape parameter $\xi_{u_{0}}$ and scale parameter $\sigma_{u_{0}}$. Therefore, the principle for selecting the threshold is to choose $u_{0}$ such that the graph of the average excess function fluctuates near a straight line for $u_{0}>0$. However, in practical engineering applications, the graph of the average excess function is rarely perfectly linear, and the threshold selection is subjective. Therefore, the optimal threshold interval can be selected, and the optimal threshold can be determined based on three fitting test criteria: root-mean-square error, correlation coefficient, and coefficient of determination. After determining the optimal threshold, the corresponding shape and scale parameters can be calculated using maximum likelihood estimation.

To further determine the optimal threshold, three commonly used testing criteria are selected to evaluate the degree of closeness between the distribution curve of the exceedance data and the theoretical distribution curve: probability plot correlation coefficient (PPCC), coefficient of determination ($R^{2}$), and root-mean-square error (RMSE), defined as:

(18) $$PPCC=\frac{\sum_{i=1}^{n}\left(x_{i}-\bar{x})(y_{i}-\bar{y}\right)}{\sqrt{\sum_{i=1}^{n}{\left(x_{i}-\bar{x}\right)}^{2}\sum_{i=1}^{n}{\left(y_{i}-\bar{y}\right)}^{2}}}$$

(19) $${\;R}^{2}=1-\frac{\sum_{i=1}^{n}{\left(x_{i}-y_{i}\right)}^{2}}{\sum_{i=1}^{n}{\left(x_{i}-\bar{x}\right)}^{2}}$$

(20) $$RMSE=\sqrt{\frac{1}{n}\sum_{i=1}^{n}{\left(x_{i}-y_{i}\right)}^{2}}$$

where $x_{i}$ is the actual value of the probability density function of the monitoring sample, $y_{i}$ is the estimated value of the GPD fit, and $\bar{x\;}$ and $\bar{y}$ are the means of $x_{i}$ and $y_{i}$, respectively.

It is first necessary to standardize the above three indicators to consider them comprehensively. Equation (21) is used to calculate the positive test indicator, meaning that the better the fitting effect, the larger the test indicator value. Equation (22) is used to calculate the inverse test indicator, which means that the better the fitting effect, the smaller the test indicator value. Then, the correlation matrix of each standardized indicator is calculated, and principal component analysis is performed on the correlation matrix. The first principal component is selected as the comprehensive indicator, and the optimal threshold is selected based on the size of the comprehensive indicator after sorting:

(21) $$a_{ij}=\frac{x_{ij}-min\;x_{ij}}{max\;x_{ij}-min\;x_{ij}}$$

(22) $$a_{ij}=\frac{max{\;x}_{ij}-x_{ij}}{max{\;x}_{ij}-min{\;x}_{ij}}$$

### 2.4. Temperature Effect Prediction Based on the SARIMA Model

#### 2.4.1. Autoregressive Moving Average (ARMA) 

Assuming that $\left\{x_{t}\right\},t=1,2,\cdots ,n$ is a stationary time series with a zero mean, the observed value $x_{t}$ at time *t* can be linearly estimated using the previous *p* observations, denoted as AR(p), as shown in the following equation [36]:

(23) $$x_{t}=\varphi_{1}x_{t-1}+\varphi_{2}x_{t-2}+\cdots +\varphi_{p}x_{t-w}+e_{t}$$

In this equation, $\varphi_{i}(i=1,2,\cdots ,p)$ represents the autoregressive coefficients, $e_{t}$ represents the error term, and p represents the order of the autoregressive model.

The observed value $x_{t}$ of the above time series at time *t* can also be represented as a linear combination of the *q* previous prediction errors, denoted as MA(*q*):

(24) $${\;x}_{t}=e_{t}-\theta_{1}e_{t-1}-\theta_{2}e_{t-2}-\cdots -\theta_{v}e_{t-v}$$

where $\theta_{i}(i=1,2,\cdots p)$ are the moving average coefficients, and q is the order of the autoregressive model:

(25) $$x_{t}=\varphi_{1}x_{t-1}+\varphi_{2}x_{t-2}+\cdots +\varphi_{w}x_{t-w}+e_{t}-\theta_{1}e_{t-1}-\theta_{2}e_{t-2}-\cdots -\theta_{v}e_{t-v}$$

which can be abbreviated as:

(26) $$\varphi (B)x_{t}=\theta (B)e_{t}$$

where $\varphi (B)=1-\varphi_{1}B-\varphi_{2}B^{2}-\cdots -\varphi_{p}B^{p}$, $\theta (B)=1-\theta_{1}B-\theta_{2}B^{2}-\cdots -\theta_{p}B^{p}$, and *B* is the backward shift operator.

The ARMA model can describe the relationship between stationary time series data without an external input and can be used for dynamic data prediction.

#### 2.4.2. SARIMA model

The seasonal autoregressive integrated moving average (SARIMA) model is developed based on the ARMA model. The premise of using the ARMA model for prediction is that the time series is a stationary random process with a zero mean. SARIMA can eliminate trend and seasonality in the time series by successive and seasonal differencing, transforming non-stationary series into stationary ones.

The SARIMA model is generally represented as SARIMA(*p*,*d*,*q*)(*P*,*D*,*Q*,*s*), where *p* is the order of the autoregressive component, *d* is the order of non-seasonal differencing, *q* is the order of the moving average component, *P* is the order of the seasonal autoregressive component, *D* is the order of seasonal differencing, *Q* is the order of the seasonal moving average component, and *s* is the length of the seasonal period. The formula is as follows [36]:

(27) $$\Phi_{P}(B^{s})\varphi (B)\nabla_{s}^{D}\nabla^{d}x_{t}=\Theta_{Q}(B^{s})\theta (B)e_{t}$$

where $x_{t}$ is the observed value at time *t* in the non-stationary time series $\left\{x_{t}\right\}$, *s* is the seasonal period length, *d* is the order of differencing applied for achieving stationarity, $\nabla_{s}^{D}$ and $\nabla^{d}$ denote the *D*th order seasonal difference operator and the *d*th order difference operator, respectively, and $\Phi_{P}(B^{s})=1-\Phi_{1}B^{s}-\Phi_{2}B^{2s}-\cdots -\Phi_{P}B^{Ps}$, $\Theta_{Q}(B^{s})=1-\Theta_{1}B^{s}-\Theta_{2}B^{2s}-\cdots -\Theta_{Q}B^{Qs}$, $\nabla_{s}^{D}={\left(1-B^{s}\right)}^{D},\;and\;\nabla^{d}={\left(1-B\right)}^{d}$. 

In this article, the Akaike Information Criterion (AIC) [37] is used to select the model order, and its expression is:

(28) $$AIC=Nln(\sigma_{e}^{2})+2p$$

where *p* represents the number of independent parameters in the model, *N* is the length of the time series, and $\sigma_{e}^{2}$ is the variance of the model residuals. The optimal order of the model is obtained when the *AIC* takes the minimum value.

## 3. Implementation of the Prediction and Early Warning

### 3.1. Experiment Design and Data Collection

**Figure 2:**
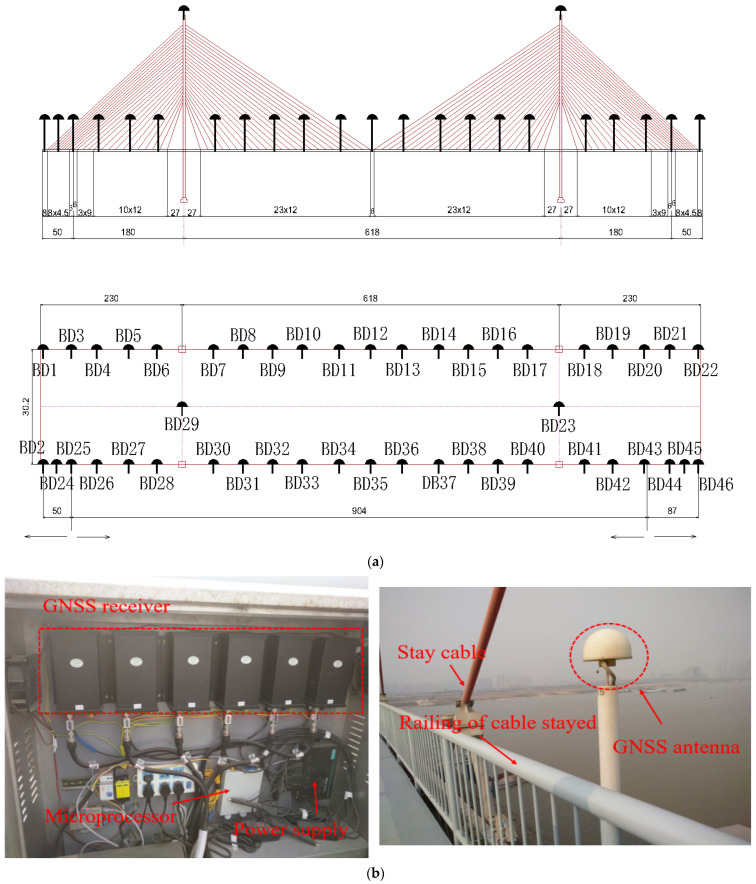
Layout of deflection monitoring points on a large cable-stayed bridge in Wuhan. (**a**) Layout of deflection monitoring points. (**b**) Beidou positioning receiver.

A case study of a cross-river cable-stayed bridge in Wuhan is carried out. The main bridge is a composite cable-stayed bridge structure of double tower double cable plane steel box girders and prestressed concrete box girders. The main pier foundation is the high pile cap foundation of the self-floating suspension box cofferdam. The main bridge is 2458 m long, the main span is 618 m, the approach bridge is 1128.38 m long, and the net width of the bridge deck is 26.5 m. The main tower is a diamond-shaped structure. The stay cables are made of high strength galvanized steel wire with a diameter of 7 mm and wrapped with a high-density polyethylene (HPE) protective layer. There are 48 pairs of cables in the upstream and downstream, and a total of 192 stay cables in the whole bridge. As shown in Figure 2, the Beidou bridge monitoring points are designed at the key parts of the bridge. The monitoring range of the cable-stayed bridge (main bridge) is 0–5 main piers, with a total of 24 sections and 46 measuring points. The sensor adopts an M300 GNSS receiver. The main parameters of the sensor are shown in Table 1.

**Figure 3:**
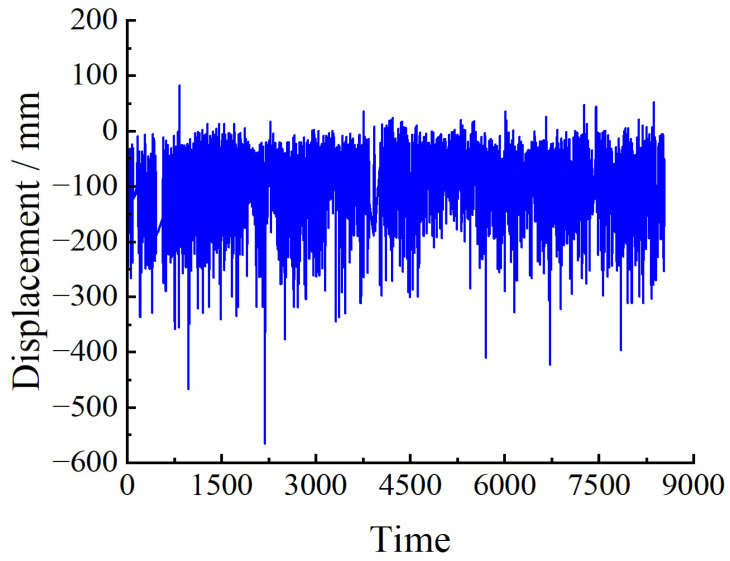
BD12 deflection monitoring data.

The monitoring data are collected once an hour. One year of data is used as the sample for setting the baseline threshold in this paper. There are missing data due to external interference in the actual monitoring operation, so relatively complete monitoring data were selected for each month to ensure continuous one-year data. Therefore, monitoring data from 1 July 2020 to 30 June 2021 were chosen to study the setting of the baseline threshold. Figure 3 shows the down-deflection monitoring data of monitoring point BD12 at each time interval from 1 July 2020 to 30 June 2021.

The VMD (variational mode decomposition)–SVD (singular value decomposition) method was used to separate temperature effects from the monitoring data, and GPD extreme value analysis was used to set the baseline threshold. The SVD is used to denoise the measured monitoring data. The sampling time is long, and the annual temperature difference effect is necessary to be considered. Therefore, the first six singular values are retained. In addition to the dominant frequency corresponding to the daily temperature difference effect with a daily cycle, the denoised data also have a dominant frequency approaching zero. In this dominant frequency, the annual temperature difference effect and the long-term deflection are included. Because the frequencies of the two are almost coincident, the number of modal decomposition K can be set to 2 when VMD is performed.

**Figure 4:**
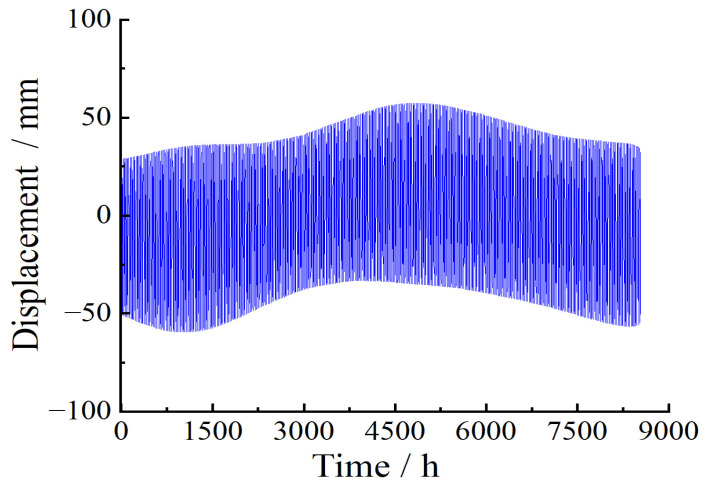
Deflection difference before and after temperature effect separation.

**Figure 5:**
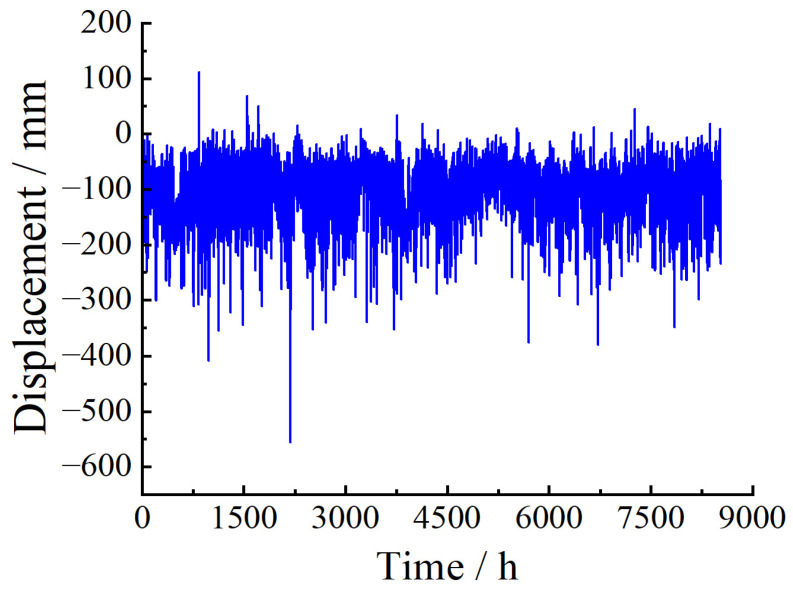
Deflection monitoring data after temperature effect separation.

The temperature effect after separation by the VMD–SVD method is shown in Figure 4. In order to show the separation effect more clearly, the temperature effect is removed from the monitored deflection data, as shown in Figure 5. The temperature effect of separation conforms to the periodic characteristics of the temperature effect. The separation results reflect the seasonal variation in the effect of temperature on the structure. Figure 5 is the deflection data after removing the temperature effect.

### 3.2. Determination of Baseline Threshold 

**Figure 6:**
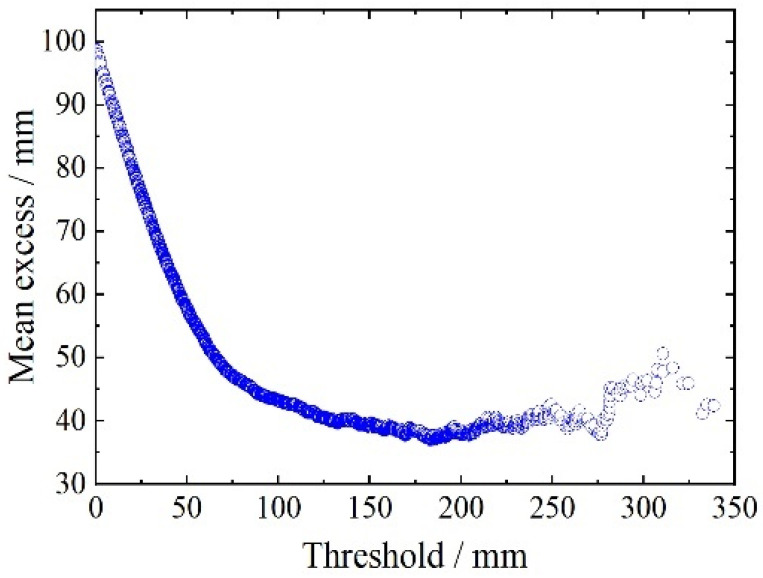
Average excess function of mid-span deflection.

A warning reference threshold is set according to the GPD extreme value analysis theory described earlier. First, the threshold of the monitoring sample is determined. The plot of the average excess function is shown in Figure 6. The figure shows that when the threshold exceeds 175 mm, the average excess function is linearly related to the threshold. However, because of the distortion caused by the tail data, the slope of the graph after 175 mm shows some fluctuations. Therefore, a threshold interval [175, 300] is chosen.

**Figure 7:**
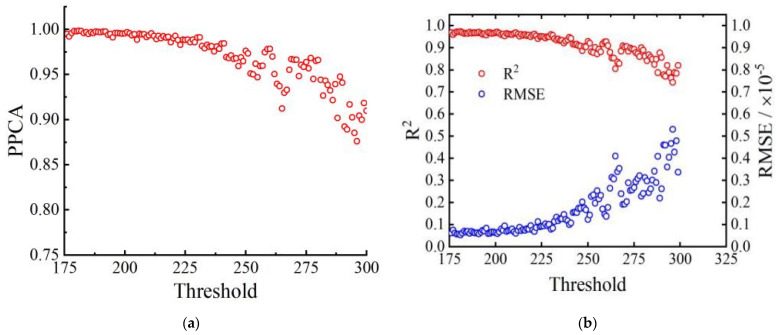
Correspondence between alternative thresholds and various verification indicators. (**a**) Correlation coefficient test, (**b**) coefficient of determination test and root-mean-square error test.

The GPD distribution parameters are estimated using maximum likelihood estimation for each candidate threshold. The expressions for the GPD distribution are obtained, and PPCC, $R^{2}$, and REMS are used to perform goodness-of-fit tests on the GPD distributions at each threshold. The relationship between the thresholds and the goodness-of-fit test indicators is shown in Figure 7.

**Figure 8:**
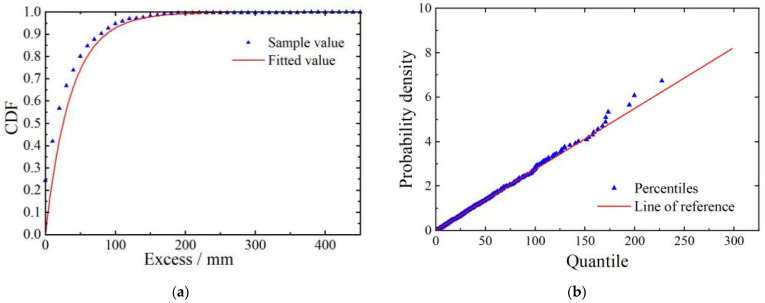
Fitting effectiveness test. (**a**) Cumulative probability density function graph. (**b**) Q–Q plot.

According to Table 2, the threshold ranked first is the optimal threshold, and its value is 181. Based on the optimal threshold, the shape and scale parameters of the GPD are estimated using the maximum likelihood method and are determined as 0.0447 and 35.8208, respectively. Cumulative probability density plots and Q–Q plots are drawn to more intuitively assess the goodness of fit between the exceedance data and the GPD distribution, as shown in Figure 8.

**Figure 9:**
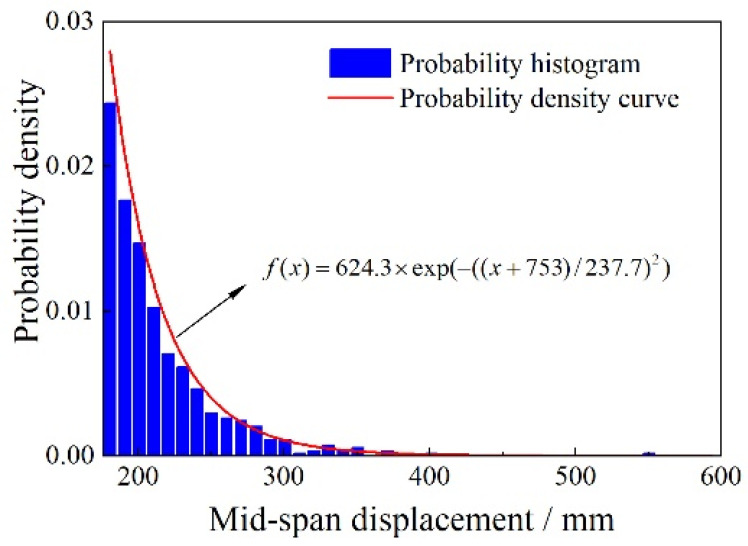
Probability density function.

Figure 8 shows that the GPD fitting curve and the cumulative distribution function of the exceedance data overlap significantly. In addition, the data points in the Q–Q plot are uniformly distributed around the fitting line, indicating a good fit. With the shape and scale parameters determined, the probability density function of the GPD is shown in Figure 9. Based on a 95% confidence level within a 100-year return period, the baseline threshold for mid-span deflection is estimated to be 504.914 mm.

### 3.3. Prediction of Temperature Effects 

**Figure 10:**
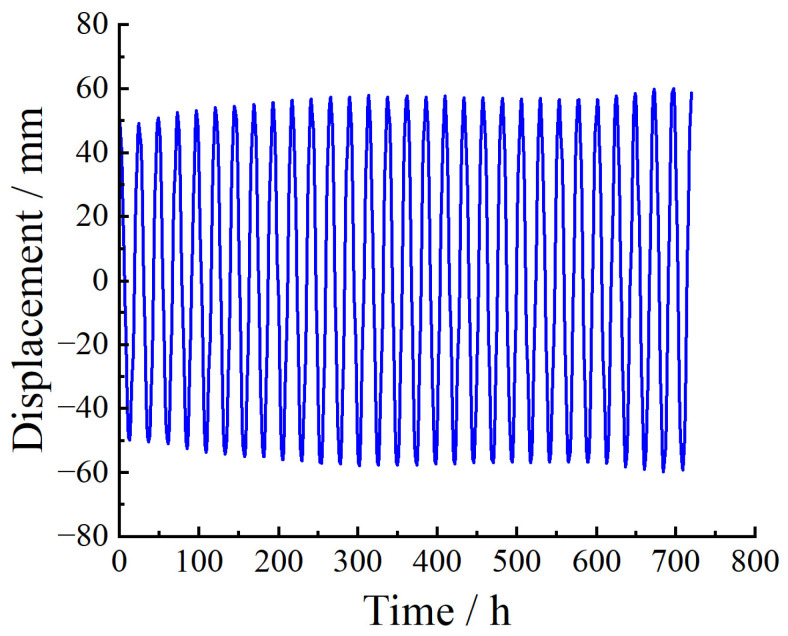
Temperature effects at the BD12 measurement point in June 2021.

**Figure 11:**
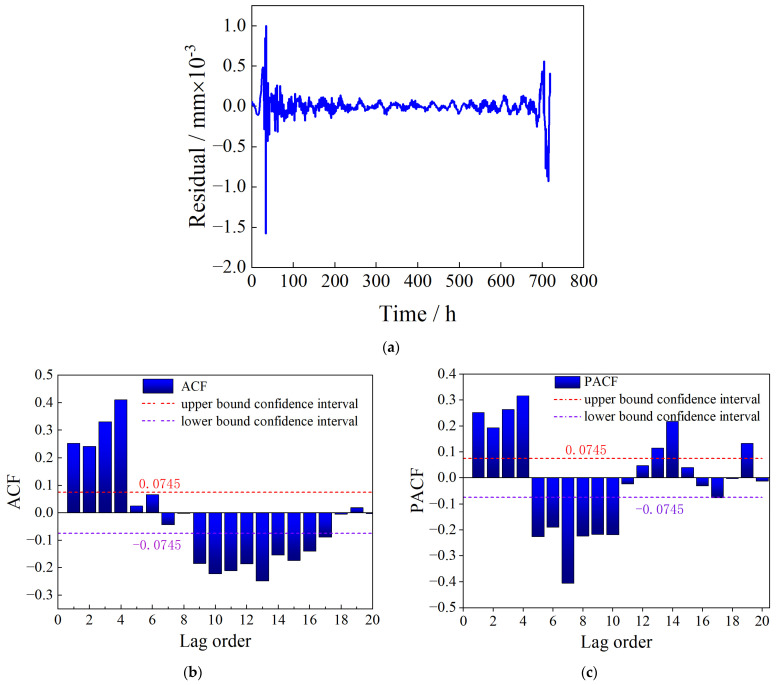
The white noise test of the model M residuals. (**a**) Time series plot of the residuals $e_{t1}.$ (**b**) Autocorrelation plot (ACF). (**c**) Partial autocorrelation plot (PACF).

When predicting temperature effects, the previous month’s 720 data points were used as training data to iteratively predict the next day’s temperature effects. Figure 10 shows the temperature effects (including daily and yearly temperature variations) at the BD12 measurement point from 1 June to 30 June 2021. It is evident from Figure 9 that the temperature effects exhibit noticeable periodicity with a period of 24 h. Therefore, SARIMA was considered for modeling and prediction. After performing first-order non-seasonal differencing and first-order seasonal differencing on the original data and using the Augmented Dickey–Fuller (ADF) test [38] to determine the stationarity of the differenced sequence, the output *p*-value was 0.0122, which is less than 0.05, indicating that the sequence is significantly stationary. Using the AIC criterion to find the remaining four parameters of the optimal SARIMA model, the search result was SARIMA(3,1,3)(0,1,3,24), denoted as model M. The residuals $e_{t1}$ of model M were tested for white noise, and the time series plot, autocorrelation plot (ACF), and partial autocorrelation plot (PACF) of $e_{t1}$ are shown in Figure 11.

As shown in Figure 11a, there is a large fluctuation in the residual value at the beginning and end. It is found that the residual fluctuation has the phenomenon of fluctuation agglomeration: the fluctuation is small in some periods and becomes large in some periods. In general, the variance of non-stationary time series models not only changes with time but also sometimes changes dramatically, showing the characteristics of ‘volatility clustering’, that is, the variance is relatively small in some periods and relatively large in other periods.

Figure 11 demonstrates that there are many autocorrelation coefficients and partial autocorrelation coefficients of the model M residuals that exceed the 95% confidence interval, where −0.075 to 0.075 is the confidence interval within which the 95% guarantee rate is met. Furthermore, the Ljung–Box test [39] was performed on the residuals $e_{t1}$, and the white noise test result shows a *p*-value of 0, which is less than 0.05, indicating rejection of the null hypothesis that there is no autocorrelation among the data, and thus $e_{t1}$ is non-white noise. There is still valuable information in $e_{t1}$, which requires further optimization of the prediction model and second-order prediction of the residuals $e_{t1}$.

**Figure 12:**
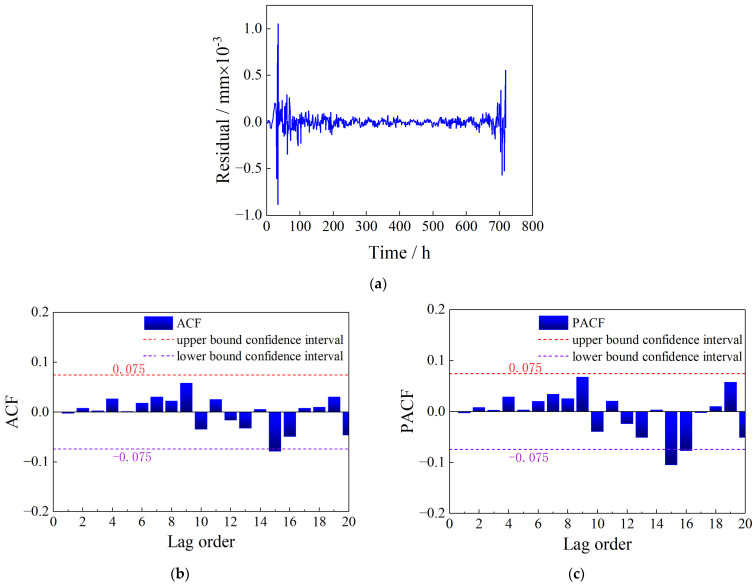
The white noise test of the model $M_{e_{t1}}$ residuals. (**a**) Time series plot of residual $e_{t2}$. (**b**) ACF plot. (**c**) PACF plot.

There is no apparent periodicity or trend in the residuals $e_{t1}$, so ARMA is considered for modeling. The ADF test was performed on $e_{t1}$, and the resulting *p*-value was 0.001, which is less than 0.05, indicating that the residual $e_{t1}$ is a stationary time series and meets the requirements for ARMA modeling. The optimal ARMA model parameters were searched using the AIC criterion, and the optimal result was found to be ARMA(5,10), denoted as model $M_{e_{t1}}$. Similarly, the white noise test was performed on the model $M_{e_{t1}}$ residuals $e_{t2}$, and the time series, ACF, and PACF plots of $e_{t2}$ are shown in Figure 12.

According to Figure 12, most of the autocorrelation and partial autocorrelation coefficients of the residual $e_{t2}$ are within the confidence interval. Although the 10th, 11th, and 15th lags exceed the confidence interval, this may be due to chance factors. The Ljung–Box test of the residual $e_{t2}$ shows a white noise statistic *p*-value of 0.2391, greater than 0.05, indicating acceptance of the null hypothesis that there is no autocorrelation between the data. That is, $e_{t2}$ is a white noise, indicating that model $M_{e_{t1}}$ fits the data $e_{t1}$ well.

**Figure 13:**
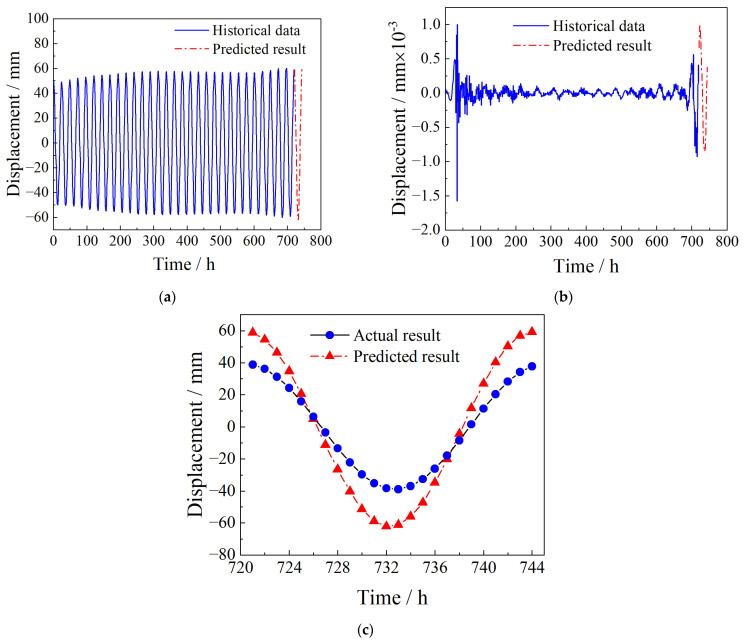
Autoregressive model prediction results. (**a**) Predicted results of model M. (**b**) Predicted results of model $M_{e_{t1}}$. (**c**) The final prediction result after one step.

The final prediction result P(t) is composed of two parts: the prediction result $P_{1}\left(t\right)$ of model M and the prediction result $P_{2}(t)$ of model $M_{e_{t1}}$, $P(t)=P_{1}(t)+P_{2}(t)$, as shown in Figure 13. The trend of the predicted values in Figure 13 is consistent with the true values, and the amplitude range is close. According to the predicted value and the real value, $R^{2}$ is equal to 0.9932.

**Figure 14:**
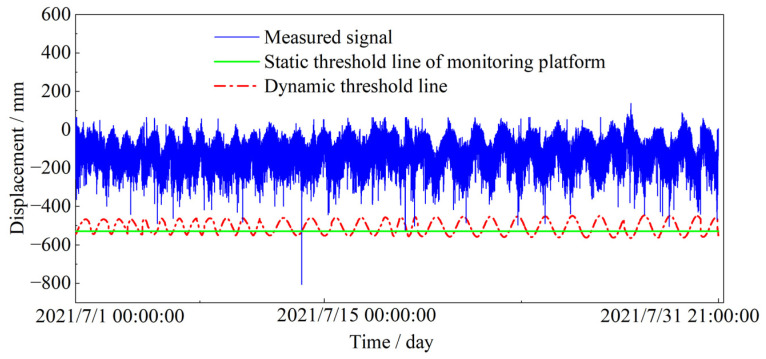
Comparison of static threshold and dynamic threshold.

As is shown in Figure 14, the predicted results are the temperature effect of monitoring point BD12 on 1 July 2021. The autoregressive analysis is repeated to achieve the temperature effect prediction for the whole of July iteratively, and the baseline threshold is dynamically corrected in turn. Figure 14 shows the dynamic threshold monitoring of the measuring point in long-span cable-stayed bridge in July. In which the dynamic threshold line is interpolated with cubic splines, and the sampling frequency is 1 Hz.

As is shown in Figure 14, there are only 10 data exceeding the static threshold, and by analyzing the data of different temperature time periods in Figure 3, it is found that the deflection of the bridge is greater in the high temperature time period than in the low temperature time period, so there will be a problem of alarm omission when utilizing the static threshold monitoring. Dynamic threshold monitoring can realize real-time adjustment of the threshold value according to the temperature change, which overcomes the error caused by seasonal temperature change. The dynamic threshold monitoring method in this paper is applied to health monitoring, and the results are shown in Table 3. In addition to the significant over-threshold alarm detected at 04:26:26 on 4 July 2021, local mutations at other time points can also be effectively monitored. A total of 170 over-threshold alerts were detected at other time points.

## 4. Discussion

When using the traditional interval extreme value method to fit the extreme value data, because this method cannot make full use of the extreme value information contained, it has certain limitations. In contrast, the GPD method is based on the over-threshold method to select the extreme value, which overcomes the problem that the extreme value information is not fully utilized. However, the selection of the threshold interval is subjective, which may lead to the deviation of the fitting results. In order to further improve the accuracy of the model, the optimal threshold can be determined by combining the root mean square error, correlation coefficient, and determination coefficient of the three fitting test criteria, so as to more accurately reflect the characteristics of the data.

In the construction of the SARIMA bridge temperature effect model, the selection of model parameters directly affects the fitting effect. When the order of p and q is selected by ACF and PACF, there will be a variety of possible results to meet the requirements. By using the AIC criterion, the model parameters can be determined more accurately. At the same time, the temperature effect correction is carried out on the basis of the reference threshold to realize dynamic monitoring and make the setting of the monitoring threshold more reasonable.

As is shown in Figure 14, the dynamic threshold warning will be adjusted in real time with the change in temperature. When the fixed threshold is used for early warning, it will not be dynamically adjusted, and the phenomenon of misstatement and omission will occur. On 4 July 2021 at 04:26:26, the static warning method detected 10 alarms, while no alarms occurred in other time periods. When using the dynamic early warning method, in addition to the 10 alerts of the static early warning. As shown in Table 3, it shows the number of alarms at different times by using the dynamic early warning method. A total of 51 alerts were detected in the 29 July 2021 11:13:31 time period. A total of 35 alarms were detected at 17:35:22 on 18 July 2021. There were 27 alarms detected at 12:19:19 on 24 July 2021. These alerts are all because the temperature effect is considered, and the structural anomalies can be effectively detected by the warning threshold line corrected by the temperature effect, the dynamic early warning effect has been significantly improved.

In practical applications, the static early warning method takes the real-time response of the bridge as the early warning index, including cable force and deflection. However, these static warning systems adopt a constant warning threshold or limit interval, which cannot change with the temperature effect. Through the discussion of three cases, Fan et al. [27] verified that the previous static early warning often had misreporting, while the dynamic early warning could change accordingly according to the temperature effect and have better mobility. The dynamic threshold can be dynamically adjusted according to real-time data and historical data to meet the threshold requirements in different situations. The SARIMA model can find out the law of data variation by learning and analyzing historical data to predict the future threshold more accurately. It reduces the possibility of mis-warning of the early warning system. 

According to the comparison between the predicted value and the actual value in Figure 13c, it can be seen that the two trends are the same, the amplitude range is close, and the relative error is 0.9932, indicating that the prediction results of the SARIMA model are accurate. Based on this, the whole of July is predicted. On this basis, the temperature effect of the reference threshold is corrected. As shown in Figure 14, using the dynamic threshold monitoring system, in addition to the 10 warnings around 4 July 2021 04:26:26 there are 170 warnings at other times. The number of alarms increased by 17 times, which greatly improved the safety and accuracy of the early warning system. Compared with static threshold monitoring, the dynamic threshold is adjusted in real time according to the change in ambient temperature. When the dynamic threshold is used to monitor the cable-stayed bridge, it has stronger adaptability and can also achieve effective monitoring of local mutations in other periods. 

When the dynamic threshold is used to monitor the cable-stayed bridge, real-time dynamic monitoring can be realized according to the change in ambient temperature, which has higher maneuverability than static threshold monitoring. However, this method also has some limitations. The residuals are small in some periods and large in other periods. This phenomenon shows that the model residual has a heteroscedasticity effect. When there is a heteroscedasticity effect, it is necessary to correctly handle the heteroscedasticity to make the estimator of the regression parameters more significant, so as to avoid the adverse effects of heteroscedasticity on the time series model and improve the prediction accuracy of the model.

## 5. Conclusions

Based on the real-time GNSS monitoring data of the bridge, the dynamic adjustment of the temperature effect on the basis of the GDP prediction baseline threshold line is proposed to realize the dynamic monitoring of the cable-stayed bridge. The main conclusions contents of this article are as follows:

(1) Based on the bridge GNSS real-time monitoring data, a dynamic early warning method for the existing cable-stayed bridge safety service monitoring platform is proposed. The static threshold monitoring process has been omitted due to the weather, while the dynamic threshold monitoring system can realize the dynamic adjustment of the threshold according to the temperature change, in addition to the significant over-threshold alarm detected at 04:26:26 on 4 July 2021, local mutations at other time points can also be effectively monitored. A total of 170 over-threshold alerts were detected at other time points, and its monitoring results are 17 times higher than the static threshold. And, the dynamic monitoring results are consistent with the manual inspection.
(2) When the traditional interval extreme value method is used to fit the extreme value data, the extreme value information contained cannot be fully utilized. GPD is based on the threshold method to select the extreme value, which overcomes the disadvantage that the extreme value information is not fully utilized. The selection of the threshold plays a decisive role in the fitting effect. Therefore, based on a variety of fitting test indicators, principal component analysis is used to obtain a comprehensive indicator to determine the optimal threshold. After determining the optimal threshold, the corresponding shape parameters and scale parameters are obtained by maximum likelihood estimation. The cumulative product probability density diagram and Q-Q diagram show that the extreme values are well fitted.
(3) The GNSS monitoring system has a total of 46 measuring point data, and the monitoring data of the mid-span BD12 measuring point are selected as the research object. In order to realize the real-time dynamic monitoring of bridge GNSS, the temperature effect of the historical monitoring data of last month is used as the training sample. SARIMA is used to model the training samples to predict the temperature effect on the next day and dynamically adjust the baseline threshold. In the white noise test of the residual, if the residual is not white noise, a second prediction is required until the residual meets the white noise requirements. The results show that the relative error between the predicted value and the true value is 0.9932, and the prediction effect of the temperature effect is accurate.
(4) In daily monitoring, due to the interference of the environment, the equipment may not work properly, resulting in abnormal data, which may lead to errors in the analysis results. Therefore, in the follow-up study, the elimination of abnormal data can be considered.

## Figures and Tables

**Table 1:** The characteristics of M300 GNSS receiver.

Characteristics	Basic Explanation
Standard single point positioning accuracy	Single frequency: H ≤ 5 m, V ≤ 5 m (1σ, PDOP ≤ 4) Dual frequency: H ≤ 3 m, V ≤ 3 m (1σ, PDOP ≤ 4)
Static differential accuracy	Level: ±(2.5 + 1 × 10^−6^ × D) mm Vertical: ±(5 + 1 × 10^−6^ × D) mm
RTK accuracy	Level: ±(8 + 0.5 × 10^−6^ × D) mm Vertical: ±(15 + 0.5 × 10^−6^ × D) mm
E-RTK accuracy	Level: ±(200 + 1 × 10^−6^ × D) mm Vertical: ±(400 + 1 × 10^−6^ × D) mm
Signal tracking	Channel number 440 BDS global signal support
Accuracy/reliability	The speed measurement accuracy is 0.03 m/s, and the initial confidence level is >99.9%. signal recapture < 1.5 s (fast), <3 s (ordinary)
Data format	Standard NMEA-0183/Supports GPGGA
Communication protocol	RS232 serial port, TCP/IP
Electrical indicators	Material: sturdy and lightweight metal packaging size: 209 × 145 × 78 mm
Physical properties	Working temperature: −40 °C~+ 70 °C Storage temperature: −55 °C~+ 95 °C Humidity: 100% fully sealed, anti-condensation, floatable

**Table 2:** Comprehensive threshold test indicator.

Threshold	Composite Indicator	Ranking	Threshold	Composite Indicator	Ranking
181	1.1300	1	239	1.0982	64
179	1.1289	2	233	1.0976	65
180	1.1289	3	238	1.0973	66
⋮	⋮	⋮	⋮	⋮	⋮
235	1.0985	63	296	0.9336	126

**Table 3:** Dynamic threshold monitoring results.

Measurement Point	Alarm Time	Occurrence Count	Measurement Point	Alarm Time	Occurrence Count
BD12	2021-07-11 12:24:43	3	BD12	2021-07-23 12:39:40	4
BD12	2021-07-14 04:26:26	10	BD12	2021-07-24 12:19:19	27
BD12	2021-07-18 17:35:22	35	BD12	2021-07-28 12:29:02	1
BD12	2021-07-18 17:36:16	7	BD12	2021-07-29 11:13:31	51
BD12	2021-07-18 17:41:28	13	BD12	2021-07-31 11:09:28	6
BD12	2021-07-19 11:26:05	6	BD12	2021-07-31 11:10:20	5
BD12	2021-07-21 14:58:11	2			

## Data Availability

Data will be made available upon request.

## List of Alphabetical Symbols

Notation	Meaning
$H_{i}(x)$	Non-degenerate distribution function
$F_{u}(x)$	Distribution function
$x_{ij}$	Test index value
$x_{i}$	The actual value of function
μ	Location parameter
σ	Scale parameter
ξ	Shape parameter
$\varphi_{i}$	Autoregressive coefficients
$e_{t}$	Error term
w	Order of the autoregressive model
$\theta_{i}$	Moving average coefficients
v	Order of the autoregressive model
u	Threshold value
$f_{u}(x)$	Probability density function
$a_{ij}$	Standardized value
$y_{i}$	Estimated value of fitting
*p*	Autoregressive order
*d*	Non-seasonal difference order
*q*	Moving average order
P	Seasonal autoregressive order
D	Seasonal difference order
Q	Seasonal moving average order
s	Period
B	Backward shift operator
